# Palmitoylethanolamide as an Intestinal Gatekeeper: Linking Inflammation, Angiogenesis and Colorectal Cancer Interception

**DOI:** 10.3390/biomedicines14092080

**Published:** 2026-09-16

**Authors:** Irene Palenca, Giovanni Sarnelli, Giuseppe Esposito

**Affiliations:** 1 Neuropharmacology and Behavioral Science Laboratory, Santa Lucia Foundation (IRCCS Fondazione Santa Lucia), 00185 Rome, Italy; irene.palenca@uniroma1.it; 2 Department of Clinical Medicine and Surgery, University of Naples Federico II, 80131 Naples, Italy; sarnelli@unina.it; 3 Department of Physiology and Pharmacology “V. Erspamer”, Sapienza University of Rome, 00185 Rome, Italy

**Keywords:** palmitoylethanolamide, ALIAmides, intestinal barrier, mucosal immunity, PPARα, angiogenesis, HIF-1α, engineered live biotherapeutics, colorectal cancer interception

## Abstract

Angiogenesis is a major determinant of tumor growth, progression and metastatic potential, and its persistent activation is closely intertwined with chronic inflammation. This relationship is particularly relevant in malignancies that arise through a prolonged inflammation-to-carcinogenesis continuum, where pathological vascular remodeling can sustain tissue hypoxia, immune-cell recruitment and the establishment of a tumor-permissive microenvironment. Colorectal cancer (CRC), especially in inflammation-associated settings, represents a paradigmatic example in which the inflammatory–angiogenic axis contributes to disease progression and unfavorable prognosis. Accordingly, identifying well-tolerated strategies capable of restraining pathological angiogenic pressure before overt neoplasia is established may have important preventive and therapeutic implications. This perspective focuses on palmitoylethanolamide (PEA), an endogenous N-acylethanolamine and prototypical autacoid local injury antagonist amide (ALIAmide) generated on demand as part of tissue homeostasis. PEA may act within a proposed multilevel intestinal homeostatic framework by preserving epithelial barrier integrity, limiting inflammatory amplification, modulating PPARα-dependent signaling and mast-cell reactivity, and restraining pro-angiogenic pathways, with context- and compartment-dependent effects on Akt/mTOR–HIF-1α/VEGF signaling. Importantly, direct preclinical evidence now supports PEA-related chemoprevention at two complementary levels: ultramicronized PEA reduced colon cancer cell proliferation and migration and decreased preneoplastic lesions and tumors in the murine azoxymethane model, whereas NAPE-PLD-engineered *Lactobacillus paracasei* F19 (pNAPE-LP) increased local PEA and reduced tumor burden, epithelial proliferation and angiogenesis in AOM/DSS colitis-associated CRC while modulating Akt/mTOR/p70S6K–HIF-1α signaling. These findings move the PEA–CRC relationship beyond a purely hypothetical association while remaining preclinical. We therefore propose that reinforcement of PEA signaling, through supplementation, PEA-oriented pharmacological strategies or engineered local biosynthesis, deserves investigation as a cancer-interception approach aimed at maintaining angiogenic and inflammatory homeostasis before autonomous tumor biology becomes established. This concept should not be interpreted as established clinical CRC prevention or as a substitute for conventional anticancer therapy.

## 1. Introduction

Angiogenesis is a tightly regulated physiological process required for tissue repair and adaptation, but persistent activation is a hallmark of chronic inflammatory disease and cancer [1]. Chronic inflammation and neovascularization form a reciprocal feed-forward circuit: inflammatory and stromal cells release cytokines, chemokines, proteases and growth factors that activate endothelial cells and promote vessel sprouting, whereas newly formed, hyperpermeable vessels facilitate further leukocyte recruitment and sustain inflammation [1,2,3]. When this response fails to resolve, inflammatory–angiogenic coupling can become a permissive program for carcinogenesis [4]. This relationship is especially relevant to cancer, where acquisition of a pro-angiogenic phenotype supports progression from microscopic or premalignant lesions toward sustained growth. VEGF is a crucial mediator, but the angiogenic switch emerges from a broader network involving inflammatory cytokines, immune and stromal cells, extracellular-matrix remodeling and hypoxia-responsive pathways [2,3]. Chronic inflammation can therefore promote tumorigenesis not only through mutagenic and proliferative pressure, but also by progressively creating the vascular support required for tissue remodeling and tumor expansion. The inflammation–driven angiogenesis axis is particularly relevant in the colon. Persistent intestinal inflammation is a recognized driver of colorectal carcinogenesis, and experimental as well as human evidence links inflammatory signaling to VEGF/VEGFR activation and tumor-associated neovascularization [5,6]. In colitis-associated cancer, chronic inflammation up-regulates VEGFR2 signaling, whereas VEGF blockade suppresses tumor development and angiogenesis [5]. These findings identify the inflammatory–vascular interface as a plausible target for preventive pharmacology before autonomous tumor biology becomes established.

In this context, palmitoylethanolamide (PEA) emerges as an endogenous candidate of particular interest. PEA is a naturally occurring N-acylethanolamine generated on demand in response to cellular and tissue stress and is considered a prototypical autacoid local injury antagonist amide (ALIAmide) [7,8]. PPARα is one of its best-characterized targets, linking lipid sensing to inflammatory control and tissue protection [9]. PEA also occurs in selected nutritional matrices and is available in formulated products for human use [10,11,12]. The central translational question for colorectal cancer (CRC) is therefore not whether PEA should be regarded as a conventional anticancer drug, but whether reinforcement of PEA signaling can help disrupt the chronic inflammatory–angiogenic coupling that makes the intestinal microenvironment progressively permissive to carcinogenesis. This perspective integrates three levels that have largely been investigated separately: endogenous, food-associated or supplementable PEA; PPARα-linked control of barrier, immune and angiogenic signaling; and programmable intestinal PEA biosynthesis by NAPE-PLD. Here, “cancer interception” denotes intervention during an inflammatory or pre-neoplastic continuum, before autonomous invasive tumor biology is established [13]. We use the term “intestinal gatekeeper” strictly as a proposed functional metaphor for the capacity of PEA to reinforce several permissive checkpoints, epithelial integrity, intestinal mucosal immune regulation, mast-cell-associated pro-angiogenic signaling and context-dependent vascular remodeling, before chronic mucosal injury becomes self-sustaining. It is not intended as a physiological classification or as an oncogenetic “gatekeeper” in the classical sense used for tumor-suppressor genes such as APC or TP53 [14]. Importantly, the CRC component of this framework is supported by direct murine evidence at two complementary levels. Exogenously administered ultramicronized PEA reduced preneoplastic lesions and tumors in azoxymethane-induced colon carcinogenesis [15], whereas pNAPE-LP reduced tumor burden in AOM/DSS-associated CRC, suppressed epithelial proliferation and angiogenesis, increased colonic PEA and modulated Akt/mTOR/p70S6K-HIF-1α signaling [16]. Thus, the proposed PEA–CRC relationship is not based solely on extrapolation from colitis or on indirect PEA-related compounds. What remains prospective is the integration of these findings into a general cancer-interception strategy and, above all, their translation to human CRC prevention.

## 2. PEA as an Endogenous ALIAmide, Food-Derived Lipid and Supplement

The dual endogenous–exogenous nature of PEA is central to its translational interest. Endogenously, PEA is synthesized and degraded locally according to tissue demand and participates in autoprotective responses that restrain excessive immune-inflammatory activation [7,8]. Historically, PEA has been identified in nutritional sources, including soy lecithin, egg yolk and peanut meal, and reviews continue to describe it as a naturally occurring food-associated lipid mediator [10]. Its occurrence in foods should not be equated with pharmacological dosing: dietary exposure is variable, quantitative intake is poorly defined and there is no evidence that ordinary food consumption reproduces tissue concentrations achieved with formulated products. Its nutritional occurrence is therefore best viewed as biological context rather than as a validated dietary prevention strategy. PEA-containing products are available, depending on jurisdiction, as nutraceuticals, food supplements or foods for special medical purposes, and micronized or ultramicronized formulations have been developed to improve oral pharmaceutical performance [10,11]. Available clinical data indicate a generally favorable tolerability profile, while preclinical toxicology of micronized PEA has not identified mutagenic or genotoxic signals under the tested conditions [11,12]. These observations are relevant to prevention but do not establish safety for years-long administration in otherwise healthy or high-risk populations. Long-term preventive use would require dedicated prospective surveillance and cannot be inferred from short- or medium-term therapeutic studies.

This distinction is important for the proposed gatekeeper framework. The term is used here to describe homeostatic modulation rather than constitutive suppression of physiological immune or vascular functions. PEA fits this concept because its biology is dominated by modulation rather than indiscriminate blockade. In experimental intestinal inflammation, orally administered PEA reduces inflammatory injury and permeability through a receptor network that includes PPARα [17], while PEA can prevent or reverse cytokine-induced epithelial hyperpermeability in vitro through PPARα-dependent mechanisms [18]. The breadth of this anti-inflammatory profile is supported beyond experimental colitis: PEA attenuates intestinal ischemia–reperfusion injury, including cytokine, adhesion-molecule, NF-κB and apoptotic responses, with a contribution of PPARα signaling, and its pleiotropic anti-inflammatory pharmacology has also been reviewed across trauma-associated inflammatory conditions [19,20]. PPARα is emphasized here because it anchors the barrier and anti-angiogenic evidence discussed below, although PEA pharmacology is pleiotropic and should not be reduced to a single receptor mechanism. Moreover, PPARα pharmacology is itself context-dependent, and translation of PPARα ligands across indications is influenced by ligand selectivity, pharmacokinetics and tissue-specific transcriptional responses [21].

## 3. PEA as a Multilevel Intestinal Gatekeeper

The gatekeeper concept provides a unifying, but explicitly hypothetical, framework for actions of PEA that are often discussed separately. At the epithelial level, PEA supports barrier integrity and limits hyperpermeability, thereby reducing access of luminal microbial products to the lamina propria [17,18]. At the immune level, PEA modulates mast-cell- and macrophage-associated inflammatory responses and engages PPARα, a nuclear receptor with broad effects on innate immune signaling [8,9,22]. Mast-cell control is particularly relevant because mast cells are strategically positioned at mucosal and perivascular interfaces and can couple chronic inflammation to vascular remodeling. At the vascular level, PEA has been shown to restrain inflammation-associated VEGF signaling through PPARα-dependent modulation of Akt/mTOR pathways in defined experimental settings [23,24,25]. These observations support a multilevel model, but they do not demonstrate that all effects occur simultaneously or with identical directionality in every intestinal compartment. Barrier failure increases microbial and antigenic pressure on mucosal immune populations; persistent immune activation amplifies cytokine, oxidative and metabolic stress; and these conditions can promote pathological vascular remodeling through a network that includes endothelial activation, extracellular-matrix remodeling, stromal signals and multiple inflammatory mediators in addition to VEGF. The hypoxia response requires particular nuance. Epithelial HIF-1α activation can be adaptive and barrier-protective during colitis [26,27,28,29], whereas persistent or dysregulated HIF-1α signaling in transformed or tumor-associated compartments can facilitate angiogenesis, metabolic adaptation and immune escape. Accordingly, the proposed preventive objective is not indiscriminate HIF-1α inhibition, but avoidance of a chronic tissue state in which inflammatory and neoplastic signals drive maladaptive hypoxia-responsive and pro-angiogenic activity. We therefore use “intestinal gatekeeper” as a functional descriptor for a proposed homeostatic model, not as evidence of a single master-regulatory pathway. Mast cells represent a second, complementary mechanistic arm of PEA-associated angiogenic restraint. PEA is well recognized as an endogenous modulator of mast-cell activation and degranulation rather than a nonspecific mast-cell suppressor [8]. Activated mast cells can release VEGF, FGF-2, histamine, cytokines, tryptase, matrix-remodeling enzymes and other pro-angiogenic mediators [30,31]. Thus, PEA may exert complementary anti-angiogenic effects through PPARα-linked restraint of Akt/mTOR-associated pro-angiogenic signaling and modulation of mast-cell-driven mediator release. The integrated PEA → mast-cell → angiogenesis pathway has not yet been directly demonstrated in CRC and should therefore be treated as a testable mechanistic hypothesis.

## 4. Intestinal Mucosal Immunity as an Interface of PEA Action

The gut-associated lymphoid tissue (GALT) comprises organized inductive structures, including Peyer’s patches and isolated lymphoid follicles, whereas intestinal mucosal immunity also includes dispersed immune populations in the lamina propria and epithelium [32,33]. Mast cells and many lamina propria macrophages should therefore not be considered synonymous with organized GALT. Throughout the revised perspective, we use the broader term “intestinal mucosal immunity” or “intestinal immune microenvironment” when referring to diffuse epithelial and lamina propria immune responses, reserving “GALT” for organized lymphoid structures. Within this framework, PEA may modulate determinants of mucosal immune activation through barrier preservation, mast-cell modulation, macrophage-associated inflammatory control and PPARα-dependent innate signaling [8,9,22]. Because chronic mast-cell activation can provide VEGF, proteases and other angiogenic mediators [29,30], such immunomodulation may also reduce local pro-angiogenic pressure. Direct evidence that PEA reorganizes organized GALT structures or alters CRC risk through GALT-specific mechanisms is lacking. Future studies should therefore distinguish organized lymphoid structures from diffuse lamina propria and epithelial immune compartments and directly examine macrophage and dendritic-cell phenotypes, mast-cell activation, lymphocyte subsets, cytokine networks, secretory IgA and epithelial permeability.

PEA is consequently positioned as a candidate lipid interface between barrier integrity and intestinal mucosal immune–vascular homeostasis. The proposed multilevel protection is sequential but not yet proven as one causal chain: reduced barrier leakage may decrease immune stimulation; lower maladaptive mucosal inflammation may reduce pro-angiogenic pressure; and the net effect may be a tissue state less permissive to neoplastic evolution. This remains a testable conceptual framework rather than an established physiological pathway.

## 5. From Intestinal Mucosal Inflammation to Pathological Vascular Remodeling in Colorectal Carcinogenesis

CRC is shaped not only by epithelial mutations but also by inflammatory, immune, stromal, microbial and vascular interactions. Chronic mucosal inflammation can establish a tumor-permissive microenvironment through cytokine production, oxidative stress, sustained epithelial proliferation, immune-cell reprogramming, extracellular-matrix remodeling and vascular adaptation [34]. VEGF represents an important bridge between inflammation and CRC, but angiogenesis is not VEGF-exclusive: endothelial-cell activation, immune-cell-derived cytokines and chemokines, stromal growth factors, proteases, extracellular-matrix remodeling, hypoxia-responsive pathways and signaling networks, including NF-κB, JAK/STAT, Wnt and Notch, all contribute to vascular remodeling [2,3,6,23]. Mast cells are one component of this broader network and can release VEGF, CXCL8, proteases and additional mediators [30,31]. Hypoxia is similarly context-dependent. In inflamed but non-neoplastic epithelium, HIF-1α can support barrier-protective adaptation [26,27,28,29], whereas persistent HIF-1α activity in evolving or established neoplastic tissue can facilitate metabolic adaptation, immune remodeling and VEGF-driven angiogenesis. The relevant preventive objective is therefore restraint of chronic pro-angiogenic pressure and pathological vascular remodeling, rather than global suppression of epithelial HIF-1α. The term “angiogenic switch” is best reserved for the transition toward a vascularized pre-neoplastic or neoplastic state. In this setting, PEA is conceptually distinct from a systemic VEGF inhibitor: it may help maintain barrier and mucosal immune homeostasis upstream of the signals that consolidate pathological vascular remodeling.

## 6. Complementary Mechanistic Arms for Angiogenic Restraint: PPARα-Akt/mTOR-HIF-1α-VEGF and Mast-Cell Modulation

**Figure 1:**
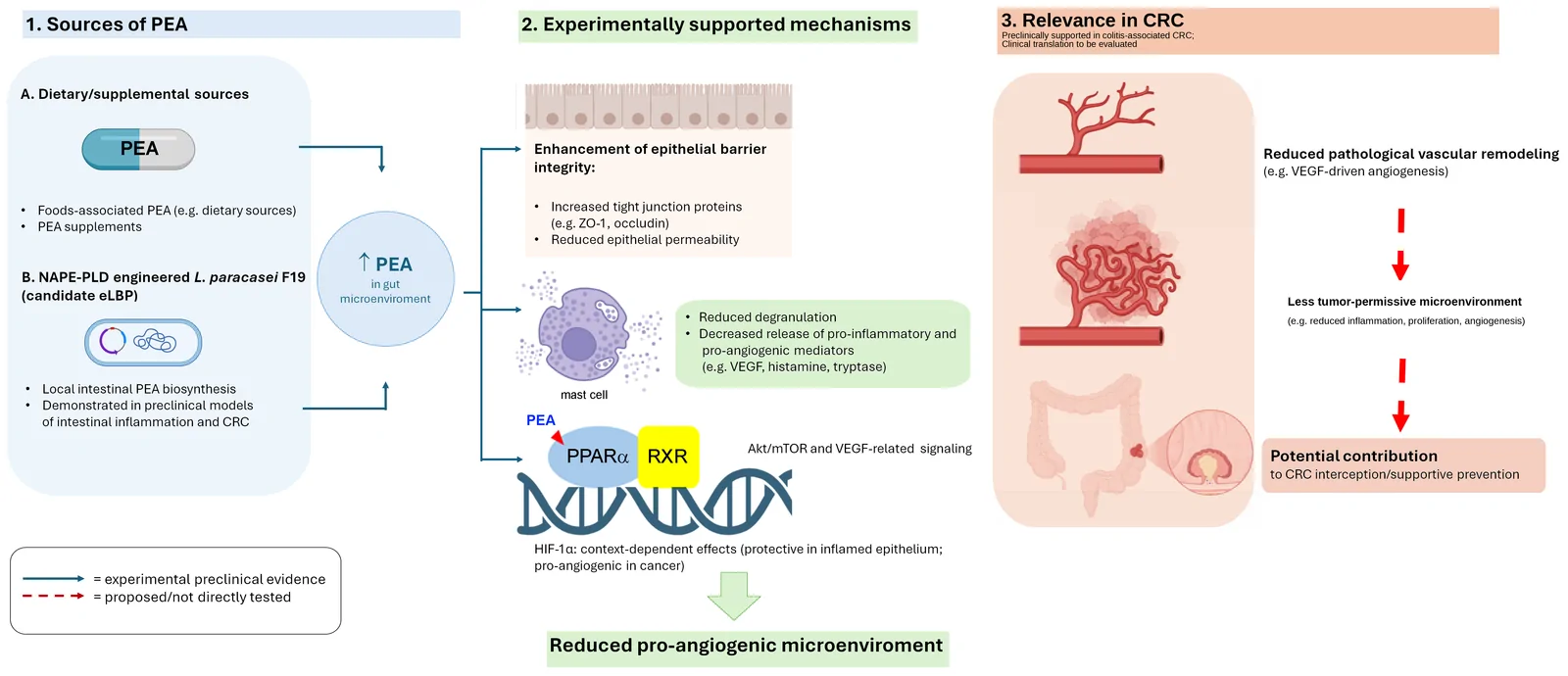
Evidence-graded model of the proposed PEA-centered intestinal homeostatic framework. Panel 1 summarizes exogenous PEA sources and local PEA biosynthesis by pNAPE-LP, a candidate engineered live biotherapeutic platform. Panel 2 summarizes experimentally supported PEA-associated mechanisms, including epithelial barrier protection, mast-cell modulation and PPARα-dependent restraint of Akt/mTOR- and VEGF-related signaling in defined preclinical settings. Direct CRC-related evidence includes chemopreventive activity of ultramicronized PEA in azoxymethane-induced colon carcinogenesis [15] and, for pNAPE-LP, reduced tumor burden, proliferation and angiogenesis with modulation of Akt/mTOR/p70S6K–HIF-1α signaling in AOM/DSS colitis-associated CRC [16]. Panel 3 places these findings within the broader CRC-interception concept. Solid arrows denote experimentally supported relationships; dashed arrows denote proposed extensions, relationships not demonstrated as a single integrated causal chain, or prospective translation beyond the available preclinical evidence. Thus, the dashed CRC-interception pathway should not be interpreted as absence of direct preclinical CRC evidence for PEA or pNAPE-LP. HIF-1α modulation is context- and compartment-dependent: epithelial HIF-1α may be barrier-protective during inflammatory injury, whereas persistent HIF-1α signaling in neoplastic compartments may support pathological angiogenesis.

Experimental evidence supports several components of this model, but the level and type of evidence differ among them. Pure PEA reduces proliferation and VEGF signaling in Caco-2 colon carcinoma cells through selective PPARα-dependent inhibition of Akt/mTOR signaling [24]. In experimental colitis and human ulcerative-colitis tissue, pure PEA also reduced inflammation-associated angiogenesis, VEGF release and neovessel formation through PPARα-dependent modulation of Akt/mTOR signaling [25]. These studies establish a mechanistic link between PEA and pro-angiogenic signaling in defined cellular and inflammatory settings, but they do not establish a universal PPARα → Akt/mTOR → HIF-1α → VEGF cascade across all intestinal cell types. Direct evidence also extends to experimental colon carcinogenesis. Pagano et al. showed that ultramicronized PEA inhibited colon cancer cell proliferation through PPARα and GPR55, induced G2/M cell-cycle arrest and DNA fragmentation, reduced tumor-cell migration, and, importantly, decreased aberrant crypt foci, polyps and tumors in the murine azoxymethane model [15]. This study therefore provides direct in vivo chemopreventive evidence for exogenously administered PEA in colon carcinogenesis, although angiogenesis was not the principal in vivo endpoint. In AOM/DSS-induced colorectal carcinogenesis, micronized N-palmitoyl-D-glucosamine (mPGA), a chemically distinct N-palmitoylated glucosamine derivative, increased colonic PEA levels while reducing pAkt/mTOR/HIF-1α signaling and VEGF-mediated angiogenesis in a PPARα-dependent manner [35]. mPGA should therefore remain classified as indirect evidence with respect to PEA itself rather than being conflated with direct PEA administration. Crucially, direct evidence is also available for engineered local PEA biosynthesis in colitis-associated CRC. In the AOM/DSS model, pNAPE-LP administered with substrate increased colonic PEA, markedly reduced mucosal damage and tumor burden, suppressed epithelial proliferation and angiogenesis, inhibited Akt/mTOR/p70S6K activation and HIF-1α expression, and beneficially remodeled tumor-associated gut dysbiosis [16]. These findings provide direct preclinical evidence that pNAPE-LP platform can influence colitis-associated colorectal carcinogenesis and its inflammatory, angiogenic signaling environment. Accordingly, it is more precise to distinguish three evidence layers: direct exogenous PEA chemoprevention in colon carcinogenesis [15]; direct pNAPE-LP efficacy in inflammation-associated CRC [16]; and indirect PEA-related evidence from mPGA for PPARα-dependent HIF-1α/VEGF restraint [35]. Direct evidence for exogenous PEA specifically against AOM/DSS-associated angiogenesis remains less developed than the broader evidence for PEA in colon carcinogenesis. Because HIF-1α can be barrier-protective in non-neoplastic epithelium [26,27,28,29], future studies should resolve epithelial, immune, stromal, endothelial and transformed-cell compartments rather than interpret bulk-tissue HIF-1α changes as uniformly beneficial or harmful. More broadly, PPARα ligands display tissue-, ligand- and context-dependent biology, and clinical translation cannot be inferred from preclinical receptor engagement alone [21]. The value of this axis is therefore not that PEA reproduces the pharmacology of a direct anti-VEGF drug, but that it may act further upstream by combining anti-inflammatory, barrier-protective and nuclear-receptor-mediated effects (Figure 1). Treatment of an established vascularized tumor remains outside the claims of this perspective.

The mast-cell arm complements this molecular network rather than duplicating it. Mast cells can amplify angiogenesis both directly, by releasing preformed or inducible pro-angiogenic mediators, and indirectly, by remodeling extracellular matrix and recruiting other inflammatory cells [30,31]. PEA-mediated control of mast-cell reactivity [8] could therefore reduce an upstream cellular source of angiogenic pressure. However, the PEA → mast-cell → CRC angiogenesis sequence has not been demonstrated as an integrated causal pathway, and the downstream consequences are likely to depend on cell type, disease stage and local microenvironment. This dual-level model is therefore proposed as a mechanistic framework for testing rather than as an established preventive mechanism (Figure 1).

## 7. From Pharmacological Chemoprevention to Cancer Interception: Why PEA Is a Particularly Attractive Molecule

The concept that chronic control of inflammation can influence colorectal neoplastic risk is not new. Epidemiological studies, randomized trials and systematic reviews indicate that aspirin and other non-steroidal anti-inflammatory drugs (NSAIDs), including COX-2 inhibitors, can reduce colorectal adenoma formation and, in selected settings, CRC incidence [36,37]. Expert guidance recognizes potential chemopreventive roles for aspirin in carefully selected individuals while advising against routine use of non-aspirin NSAIDs for average-risk prevention because of adverse events [38].

This experience provides biological proof of principle that sustained modulation of inflammatory pathways can alter colorectal neoplastic risk, but it should not be interpreted as evidence that PEA will reproduce NSAID efficacy in humans. The evidence levels remain fundamentally different: NSAID chemoprevention is supported by human epidemiological and interventional data, whereas PEA has no clinical evidence for CRC prevention. Nevertheless, the PEA field is not devoid of direct cancer-prevention evidence. Ultramicronized PEA reduced preneoplastic lesions and tumors in an AOM model [15], and pNAPE-LP reduced tumor burden and angiogenic/proliferative signaling in AOM/DSS colitis-associated CRC [16]. The comparison with NSAIDs is therefore conceptual at the clinical level, while the PEA premise is already supported by direct preclinical proof of principle. Chronic NSAID exposure can be limited by gastrointestinal bleeding and ulceration, cardiovascular events and renal toxicity [37,38]. PEA is conceptually attractive because it is an endogenous homeostatic lipid with a generally favorable current tolerability profile [11,12], but “favorable tolerability” must not be equated with proven long-term preventive safety. The rational next step is therefore not to establish whether a preclinical chemopreventive signal exists, but to reproduce it independently, define the contribution of formulation and route of delivery, compare conventional and locally biosynthesized PEA, and establish the long-term benefit–risk profile required for prevention.

Here, cancer interception is used deliberately but narrowly. Contemporary oncology defines interception as proactive disruption of cancer evolution at premalignant or very early stages [13]. In the present model, the populations of greatest future translational interest would therefore not be healthy individuals indiscriminately, but subjects or experimental systems in which chronic inflammatory injury, premalignant changes or validated CRC-risk states can be identified. The central proposition is not that PEA is a clinically established CRC-preventive intervention; rather, direct AOM and AOM/DSS data provide a preclinical basis for testing whether reinforcement of PEA-related homeostatic signaling can be developed into a supportive interception strategy capable of maintaining the mucosal inflammatory–angiogenic environment below a tumor-permissive threshold before autonomous invasive tumor biology is established [15,16].

## 8. Local PEA Biosynthesis: Why Spatial and Temporal Exposure May Matter

A critical issue in translating this proposed framework is spatial and temporal exposure. Endogenous PEA is generated locally and on demand, whereas conventional supplementation depends on formulation, luminal dissolution, absorption, metabolism and tissue distribution. Supplementation is currently the most accessible exogenous strategy, but it does not necessarily reproduce the site-restricted kinetics of autacoid biology. Equally, the available engineered-bacteria studies do not establish continuous, stable or years-long PEA production. The potential advantage of in situ biosynthesis should therefore be framed as experimentally observed local production with the possibility of repeated or spatially enriched exposure, rather than as “constant availability”. If PEA can be generated reproducibly within the intestinal environment, exposure may be enriched near the epithelial barrier and lamina propria, where mucosal immune activation and early vascular remodeling intersect. This predicted advantage is spatial pharmacology, not simply “more PEA”, and must be demonstrated by quantitative tissue pharmacokinetics rather than assumed from bacterial administration. Future studies should determine the duration and anatomical distribution of bacterially generated PEA, compare local and systemic exposure with formulated supplementation, and establish whether barrier, immune and vascular effects co-occur within the same temporal window. Until such data are available, terms such as “persistent”, “continuous” and “permanent” should be reserved for future design goals rather than used to describe the current platform.

## 9. NAPE-PLD-Engineered *L. paracasei* F19 as a Candidate Engineered Live Biotherapeutic Platform

A strategic extension of the concept is local PEA production by an engineered bacterial chassis. Although the parental organism is the probiotic strain *L. paracasei* F19, genetic modification to express a pharmacologically relevant biosynthetic enzyme moves the platform beyond conventional probiotic supplementation and into the field of candidate engineered live biotherapeutic products (eLBPs) [39,40,41,42,43,44]. Hereafter, we refer to pNAPE-LP as a candidate eLBP, while retaining the specific strain designation when discussing experimental data. Initial proof of principle for local PEA production was provided by studies showing that pNAPE-LP, administered with ultra-low palmitate supplementation, increased PEA output in vitro and intestinal PEA levels in vivo and protected mice from experimental colitis through PPARα-dependent mechanisms [45,46]. Importantly, the platform has now also been directly evaluated in AOM/DSS colitis-associated colorectal cancer. In that model, pNAPE-LP increased colonic PEA and markedly reduced mucosal damage and tumor burden; it suppressed epithelial proliferation and angiogenesis, restored p53-wt expression, inhibited Akt/mTOR/p70S6K activation and HIF-1α expression, and shifted tumor-associated dysbiosis toward a more homeostatic microbial configuration [16]. The inactivity of control pLP and palmitate alone supported dependence on engineered PEA production under the experimental conditions [16]. These CRC data substantially extend the earlier inflammatory proof of principle: the platform is no longer supported only by colitis or toxin-injury models, but by direct preclinical efficacy in inflammation-associated colorectal carcinogenesis, including angiogenic endpoints. They do not, however, establish clinical CRC prevention, efficacy against established autonomous tumors, or long-term preventive biosafety. The biochemical interpretation also requires precision. NAPE-PLD does not convert free palmitate directly into PEA. Rather, N-acylethanolamine biosynthesis classically requires upstream N-acylation of phosphatidylethanolamine to form an appropriate NAPE species, followed by NAPE-PLD-mediated hydrolysis to release the corresponding N-acylethanolamine [47]. NAPE species also occur in prokaryotic cells, but the specific NAPE-forming acyltransferase or phospholipid-remodeling activity responsible for generating the relevant N-palmitoyl substrate in the engineered F19 system was not directly identified in refs. [44,45]. Accordingly, palmitate should be interpreted as an experimentally effective precursor input that enhanced PEA output in the engineered system, not as the sole or direct substrate of NAPE-PLD. The relative contribution of bacterial phospholipid metabolism versus luminal or host-derived NAPE pools remains unresolved and should be directly quantified in future work through measurement of NAPE molecular species, flux analysis and identification of the relevant upstream acyltransferase/remodeling activity. Taken together, the available studies establish local biosynthetic activity and preclinical biological efficacy across inflammatory intestinal injury and AOM/DSS colitis-associated CRC [16,45,46]. What remains prospective is translation: predictable product output, genetic stability, physiological robustness, controlled persistence, manufacturing reproducibility and biocontainment will be required rather than indefinite bacterial colonization. These limitations are consistent with the broader eLBP field, in which gut fitness, interindividual variability, delivery, genetic stability, manufacturing and biosafety remain major barriers to translation [40,41,42,43,44,48].

Beyond direct PEA supplementation, a broader set of PEA-oriented or mechanistically convergent strategies may be considered within the same conceptual framework, but their evidentiary weight is not equivalent. Formulated ultramicronized PEA provides direct exogenous reinforcement of the endogenous ALIAmide system and has demonstrated chemopreventive activity in AOM-induced colon carcinogenesis [15]. Micronized N-palmitoyl-D-glucosamine (mPGA), by contrast, is a pharmacologically distinct compound that increased colonic PEA levels and reduced angiogenic readouts in AOM/DSS carcinogenesis [35]; it should therefore be regarded as indirect evidence for the PEA-related system rather than as direct PEA administration (Table 1). pNAPE-LP provides a second, mechanistically distinct line of direct evidence: beyond inflammatory models [44,45], pNAPE-LP reduced tumor burden, proliferation and angiogenesis in AOM/DSS colitis-associated CRC while increasing colonic PEA and modulating Akt/mTOR/p70S6K–HIF-1α signaling [16]. Adelmidrol remains a distinct ALIAmide-related compound whose anti-angiogenic effects were demonstrated in a non-CRC carrageenan-granuloma model [49]; this evidence is mechanistically convergent but indirect. Table 2 therefore separates direct exogenous PEA, direct engineered PEA biosynthesis and indirect PEA-related approaches rather than treating compounds, models and levels of evidence as interchangeable (Table 1).

## 10. Translational Priorities and Critical Caveats

Several questions must be resolved before these preclinical findings can be translated. First, the next experimental step is not simply to demonstrate CRC-related efficacy for the first time: direct chemopreventive evidence already exists for ultramicronized PEA in AOM-induced colon carcinogenesis [15] and for pNAPE-LP in AOM/DSS colitis-associated CRC [16]. These findings should now be independently reproduced and extended in harmonized designs, including head-to-head comparison of conventional PEA supplementation with engineered local biosynthesis in the same carcinogenesis model. Administration should be aligned to defined inflammatory and pre-neoplastic windows, with tumor incidence, multiplicity, dysplasia, proliferation and vascular density followed longitudinally. Second, intestinal immune endpoints must be measured rather than inferred. Organized GALT structures should be analyzed separately from the diffuse lamina propria and epithelial immune compartments, including macrophage phenotype, mast-cell density and activation/degranulation state, dendritic-cell and lymphocyte profiles, cytokine networks and IgA-related responses. These should be integrated with permeability, tissue PEA, PPARα, pAkt, pmTOR, p70S6K, compartment-resolved HIF-1α, VEGF/VEGFR and vascular markers such as CD31 or endomucin. Third, local biosynthesis should be quantitatively compared with conventional PEA supplementation to determine whether spatially enriched production provides a genuine pharmacological advantage rather than merely an alternative route of exposure. Fourth, bacterial load, palmitate exposure, NAPE substrate availability, local PEA concentration, systemic exposure and target engagement must be quantified rather than inferred. Finally, the intervention should be developed as an eLBP, with attention to genetic stability, removal of antibiotic-resistance markers, horizontal gene transfer, controlled persistence, manufacturing reproducibility and biocontainment [39,40,41,42,43,44,48]. The preventive setting raises the safety threshold because intervention may be prolonged and directed to individuals without invasive disease.

## 11. Conclusions

PEA is best positioned in this perspective not as a conventional anticancer treatment or a clinically proven CRC-preventive agent, but as a candidate homeostatic modulator for which direct preclinical chemopreventive evidence is already available. Ultramicronized PEA reduced colon cancer cell proliferation and migration and decreased preneoplastic lesions and tumors in the murine AOM model [15], while pNAPE-LP reduced tumor burden, proliferation and angiogenesis in AOM/DSS colitis-associated CRC while increasing colonic PEA and restraining Akt/mTOR/p70S6K–HIF-1α signaling [16]. These complementary findings support the biological plausibility of reinforcing PEA tone by either exogenous administration or engineered local biosynthesis. The translational interest of this strategy lies in its potential to preserve epithelial barrier integrity, temper maladaptive mucosal immune activation, modulate mast-cell reactivity and reduce pro-angiogenic signaling before autonomous tumor biology is established. Any effect on HIF-1α should be understood as context-, cell type- and disease-stage-dependent rather than uniformly inhibitory.

This framework therefore warrants investigation in experimental and clinical-risk settings characterized by persistent inflammatory injury, identifiable pre-neoplastic changes or validated CRC-risk states, in which prolonged inflammatory and angiogenic pressure can progressively create a tumor-permissive microenvironment. The rationale is to determine whether the preclinical chemopreventive effects already observed with exogenous PEA [15] and engineered local PEA biosynthesis [16] can be reproduced, mechanistically resolved and ultimately translated into a measurable reduction in human CRC risk. The NSAID experience demonstrates that inflammation-targeted colorectal prevention is clinically plausible, but it does not establish PEA efficacy [36,37,38]. mPGA provides additional, indirect evidence that enhancement of the PEA-related system can restrain PPARα-dependent HIF-1α/VEGF angiogenic signaling in AOM/DSS [35], whereas adelmidrol provides only non-CRC mechanistically convergent evidence [49]. Thus, the remaining uncertainty concerns translation and the completeness of the proposed mechanistic chain, not the existence of preclinical CRC-related efficacy. If confirmed independently and translated safely, PEA supplementation and quantitatively controlled engineered local biosynthesis could be investigated as adjunctive components of cancer-interception strategies in selected high-risk settings without substituting for conventional anticancer treatment.

## Figures and Tables

**Table 1:** Evidence map distinguishing direct preclinical PEA and engineered-PEA evidence from indirect PEA-related evidence and prospective translational hypotheses.

Proposed Link	Current Evidence	Status
PEA → intestinal barrier protection	Experimental intestinal inflammation and epithelial permeability studies [17,18]	Supported preclinically
PEA → mast-cell modulation → lower pro-angiogenic mediator pressure	PEA regulates mast-cell activation [8]; mast cells contribute to tumor/CRC angiogenesis [30,31]	Components supported; integrated CRC pathway not directly tested
PEA → PPARα/Akt/mTOR/VEGF modulation	Cellular, colitis and human UC tissue evidence [24,25]	Supported in defined experimental models
Exogenous ultramicronized PEA → reduced colon carcinogenesis	In vitro antiproliferative/antimigratory effects and in vivo reduction in aberrant crypt foci, polyps and tumors in the AOM model [15]	Direct preclinical evidence for PEA chemoprevention; angiogenesis was not the principal in vivo endpoint
mPGA-associated increase in colonic PEA tone → HIF-1α/VEGF restraint in AOM/DSS	mPGA, not exogenous PEA, increased colonic PEA and reduced pAkt/mTOR/HIF-1α/VEGF-related angiogenic readouts in AOM/DSS [35]	Indirect evidence for the PEA-related system; pharmacologically distinct from PEA
pNAPE-LP → local intestinal PEA production	Engineered-bacteria studies demonstrate increased intestinal PEA in inflammatory models [44,45] and in AOM/DSS colitis-associated CRC [16]	Directly demonstrated preclinically; upstream NAPE-generating pathway remains incompletely characterized
Engineered-bacteria-derived PEA → reduced inflammatory–angiogenic signaling	Toxin A intestinal injury [46] and AOM/DSS colitis-associated CRC, with reduced proliferation/angiogenesis and Akt/mTOR/p70S6K–HIF-1α signaling [16]	Supported preclinically in defined inflammatory and CAC models
Local PEA → organized GALT reprogramming	No direct integrated study of organized GALT structures	Not directly tested/Hypothesis
pNAPE-LP → reduced colitis-associated CRC development	Reduced tumor burden in AOM/DSS with increased colonic PEA and suppression of proliferative/angiogenic signaling [16]	Direct preclinical evidence in colitis-associated CRC
PEA-based strategies → clinical CRC interception	No prospective human CRC-prevention trials	Not clinically established

**Table 2:** Direct PEA engineered PEA-biosynthesis and mechanistically convergent strategies relevant to the proposed intestinal homeostatic/angiogenic framework.

Strategy	Relationship to the PEA/ALIAmide Framework	Evidence Relevant to Gatekeeper/Angiogenic Restraint	Translational Interpretation
Native/formulated PEA	Direct pharmacological reinforcement of the endogenous PEA/ALIAmide system	Barrier protection and reduced intestinal hyperpermeability [17,18]; mast-cell modulation [8]; PPARα-linked restraint of Akt/mTOR-VEGF signaling [24,25]; direct chemopreventive activity in AOM-induced colon carcinogenesis [15].	Direct pharmacological reinforcement of the PEA system with preclinical colon-cancer chemopreventive evidence; CRC-preventive efficacy in humans remains unproven.
Micronized/ultramicronized PEA	Formulation-based delivery of exogenous PEA; not a distinct analog	Ultramicronized PEA inhibited colon cancer cell proliferation and migration, affected cell-cycle control, and reduced aberrant crypt foci, polyps and tumors in the murine AOM model [15]. The pharmacokinetic and safety literature supports generally favorable short-/medium-term tolerability [11,12].	Direct preclinical chemopreventive evidence in colon carcinogenesis; long-term preventive safety, optimal formulation and clinical efficacy remain to be established.
Micronized N-palmitoyl-D-glucosamine (mPGA)	Indirect PEA-tone-enhancing strategy; N-palmitoylated glucosamine derivative	In AOM/DSS CRC, mPGA increased colonic PEA and reduced mucosal damage, CD31/VEGF-mediated angiogenesis and pAkt/mTOR/HIF-1α signaling in a PPARα-dependent manner [35]. The administered compound was mPGA, not PEA.	Indirect evidence for the PEA-related system; pharmacologically distinct from direct PEA administration.
pNAPE-LP	Biosynthetic reinforcement through local PEA production in a candidate eLBP; palmitate is an experimentally effective precursor input, not the direct NAPE-PLD substrate	Earlier studies demonstrated increased intestinal PEA, barrier protection and PPARα-dependent anti-inflammatory activity [45], with HIF-1α/VEGF-related effects in toxin A intestinal injury [46]. In AOM/DSS colitis-associated CRC, pNAPE-LP increased colonic PEA, reduced tumor burden, proliferation and angiogenesis, inhibited Akt/mTOR/p70S6K and HIF-1α, and remodeled tumor-associated dysbiosis [16].	Direct preclinical evidence for local PEA biosynthesis and efficacy in colitis-associated CRC; clinical interception efficacy, long-term biosafety and manufacturing/containment requirements remain unresolved.
Adelmidrol	Distinct ALIAmide-related compound included only as mechanistically convergent, indirect evidence	Adelmidrol reduced mast-cell degranulation, pro-inflammatory/pro-angiogenic mediators and neovascularization in a carrageenan-granuloma model [49], a non-CRC inflammatory angiogenesis setting.	Indirect, non-CRC evidence. These findings cannot be attributed to PEA itself and should not be used as primary evidence for PEA-mediated CRC effects.

## Data Availability

No new data were created or analyzed in this study. Data sharing is not applicable to this article.

## Abbreviations

The following abbreviations are used in this manuscript:

ALIAmide	Autacoid local injury antagonist amide
AOM/DSS	Azoxymethane/dextran sulfate sodium
CRC	Colorectal cancer
eLBP	Engineered live biotherapeutic product
GALT	Gut-associated lymphoid tissue
HIF-1α	Hypoxia-inducible factor-1α
mTOR	Mammalian target of rapamycin
NAPE-PLD	N-acylphosphatidylethanolamine-preferring phospholipase D
PEA	Palmitoylethanolamide
PPARα	Peroxisome proliferator-activated receptor-α
VEGF	Vascular endothelial growth factor
